# Conversion from vertical to tangential view using the anchor traction method in colorectal endoscopic submucosal dissection

**DOI:** 10.1055/a-2791-4809

**Published:** 2026-03-09

**Authors:** Keisaku Yamada, Masahiro Tajika, Tsutomu Tanaka, Nobuhito Ito, Akihiro Takagi, Yasumasa Niwa

**Affiliations:** 1 Department of Endoscopy, Aichi Cancer Center Hospital, Nagoya, Japan

In colorectal endoscopic submucosal dissection (ESD), achieving a tangential approach to the lesion facilitates safe and effective dissection by providing a stable view of the submucosal layer. We developed a novel traction technique using a multi-loop traction device (MLTD; Boston Scientific Co., Ltd, Tokyo, Japan) that enables traction at three points, termed the “anchor traction method” 1 2 3 . Here, we report a case in which the anchor traction method enabled safe and effective dissection by converting the endoscopic view from vertical to tangential during colorectal ESD.

**Fig. 1:**
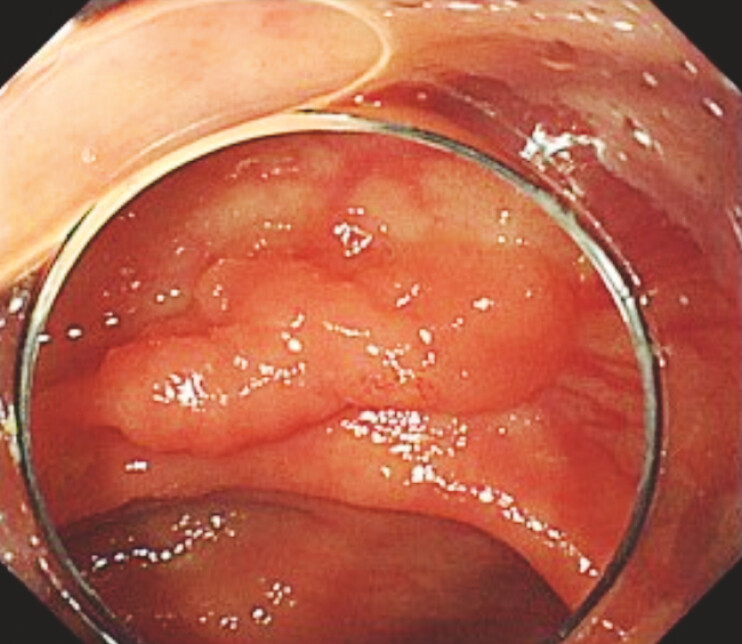
The lesion was a 20mm IIa lesion at the ascending colon.

A 68-year-old woman presented with a 20-mm IIa lesion in the ascending colon ( Fig. 1 ) and underwent ESD ( Video 1 ). After a full circumferential incision was made, submucosal dissection was attempted; however, it was difficult to proceed because the lesion was vertically confronted with the endoscope. As submucosal dissection was technically difficult, traction was applied using the anchor traction method. The middle loop of the MLTD was attached to a reopenable clip (SureClip Eco; MicroTech, Nanjing, China), and the two additional loops were then anchored to the lesion, as previously described for the anchor traction method.

An useful method for lesions that are vertically confronted with the endoscope.Video 1

**Fig. 2:**
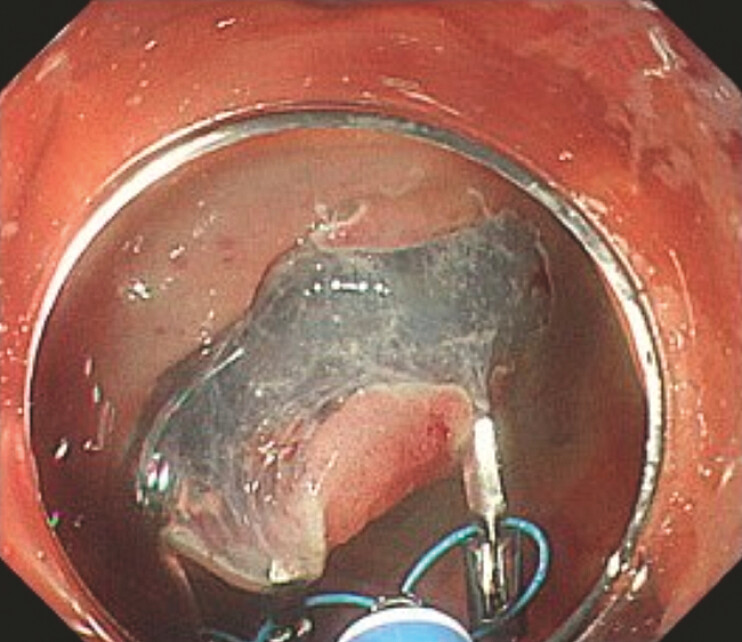
The anchor traction method converted the lesion’s orientation to a tangential angle, resulting in easier and safer submucosal dissection.

**Fig. 3:**
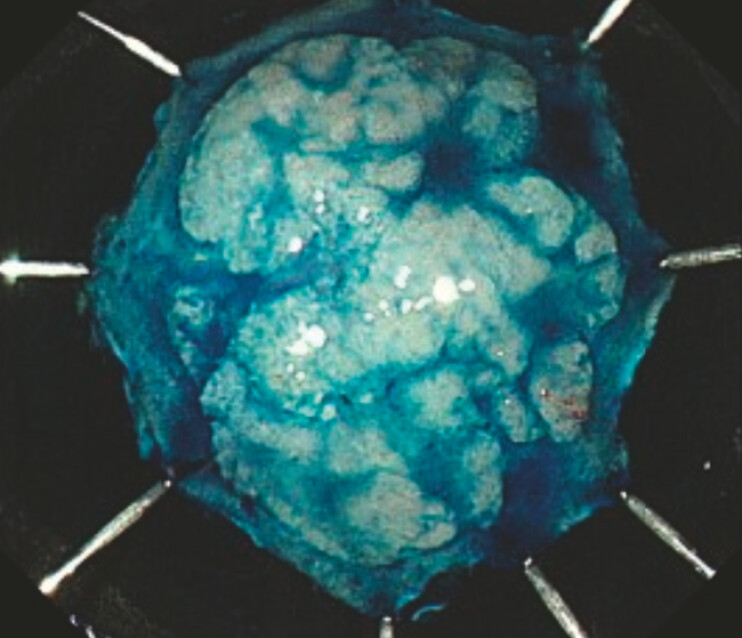
Pathological analysis revealed that the lesion was a 22 × 15 mm high grade dysplasia with negative margins.

The use of the anchor traction method converted the lesion’s orientation to a tangential angle ( Fig. 2 ), resulting in easier and safer submucosal dissection, and the en bloc resection was completed ( Fig. 3 ). In this method, three-point traction with a shorter traction length enables strong traction of the entire lesion and transforms the endoscopic approach from a vertical to a tangential view. This technique is particularly useful for lesions that are vertically confronted with the endoscope.

Endoscopy_UCTN_Code_TTT_1AQ_2AD_3AD
